# The effects of mirror visual feedback involved network priming on embodiment perception in healthy subjects: a proof-of-concept study

**DOI:** 10.3389/fnins.2026.1781002

**Published:** 2026-03-12

**Authors:** Yongxin Luo, Lin Cai, Juan Li, Jianwei Lu, Li Ding

**Affiliations:** 1 School of Rehabilitation Science, Shanghai University of Traditional Chinese Medicine, Shanghai, China; 2 Engineering Research Center of Traditional Chinese Medicine Intelligent Rehabilitation, Ministry of Education, Shanghai, China; 3 School of Sport, Exercise and Health Sciences, Loughborough University, Loughborough, United Kingdom; 4 Department of Rehabilitation Medicine, Community Health Service Center of Xianxia, Shanghai, China; 5 Department of Rehabilitation Medicine, Huashan Hospital, Fudan University, Shanghai, China

**Keywords:** mirror visual feedback, embodiment perception, brain network, rubber hand illusion, action observation

## Abstract

**Introduction:**

Mirror visual feedback (MVF) efficacy varies with individual embodiment perception.

**Objective:**

The study aimed to investigate the behavioral effects of priming via the rubber hand illusion (RHI) and action observation (AO) on embodiment perception during MVF.

**Methods:**

Twenty healthy participants were recruited. This experiment contained three rounds: MVF, RHI-MVF, and AO-MVF. At first, all the participants completed the round of MVF, and after 24 hours, they received the round of RHI-MVF or AO-MVF at a random order with an interval of 24 hours. Each round comprised two sessions, including session of simple motor tasks (SMT) and session of objective-based tasks (OBT). In addition, each session contained 5 tasks, which was repeated 10 times at a frequency of 2 seconds per time.

**Results:**

The results showed that priming of networks overlapping with MVF through RHI/AO paradigms could enhance the intensity of embodiment perception. The machine learning analysis further revealed a stronger predictive association between RHI and heightened embodiment perception compared to AO. Additionally, we found that OBT could facilitate embodiment elicitation, comparing to SMT.

**Conclusion:**

Our findings provided, which insights into modulating embodiment perception during MVF paradigms. These preliminary results might benefit future investigations therapeutic efficacy in neuro-rehabilitation.

**Clinical trial registration:**

Identifier ChiCTR2500102438.

## 1 Introduction

Mirror visual feedback (MVF) was firstly introduced by Ramachandran et al. (1992) as a strategy for the management of phantom limb pain. Since then, MVF has been recognized as an appropriate health technology for its convenient use and low cost, attracting widespread attention in the field of neuro-rehabilitation, specifically for upper limb function following stroke (Ding et al., 2019; Ramachandran and Altschuler, 2009). By reflecting the movement of unaffected limb, MVF provides the brain with virtual visual information of the affected side, which allows individuals post-stroke perceive an optical illusion that their affected limbs move normally, and rebalances the conflict of motor output and visual input after unilateral limb paralysis (Altschuler et al., 1999; Nogueira et al., 2021; Thieme et al., 2018). This illusion can generate a sense of embodiment, referring to a subjective perception of the bodily awareness, which plays a crucial role in the efficacy of MVF (Nogueira et al., 2021). Thus, variations in the perception of embodiment among individuals might lead to inconsistent therapeutic outcomes, and it is one of the neglected factors hindering the application of MVF in hospitals with lower levels or in rural areas.

Underlying mechanisms of MVF have been the focus of much interest and debate in terms of stroke rehabilitation, which might benefit for the improvement of treatment efficacy. According to the majority of studies, MVF could promote the motor recovery through activating primary motor cortex as instant neuro-modulatory effects and enhancing recruitment of ipsilateral corticospinal pathways as neuroplastic effects (Zhang et al., 2024; Ding et al., 2025). Other studies further suggested that motor relevant cortices, including premotor cortex (PMC) and supplementary motor area, are also related to neural reorganization and functional recovery due to MVF intervention (Bonnal et al., 2024; Bai et al., 2020). It is worth noting that PMC is associated with the mirror neuron system (MNS) and superior temporal gyrus (STG), another area involved in the MNS, which could be also activated by MVF (Pineda, 2008). Both areas form the network can contribute the imitation and motor relearning (Pineda, 2008). Beyond the mentioned areas, studies also reported visual and somatosensory areas, posterior parietal cortex (PPC), and cingulate cortex, revealing the impact of MVF on increasing attentional demands toward the affected side after stroke (Deconinck et al., 2015). It is well known that the precuneus, medial extension of superior PPC, is related to visuospatial information and spatial attention, which further supports the effects of MVF on attentional process via perceptual inputs (Pereira-Pedro and Bruner, 2016). The above studies suggest three mainstream hypotheses for underlying mechanisms of MVF from the perspective of brain networks, including motor network, MNS, and attentional network (Deconinck et al., 2015). Thus, we proposed to pre-activate these MVF involved networks, and to investigate the effect of this priming strategy on embodiment perception due to MVF, which might contribute to improve the efficacy and to promote the clinical application of MVF in neuro-rehabilitation. Our preliminary study demonstrated that repeated transcranial magnetic stimulation (rTMS) priming over motor process-related regions in healthy individuals contribute to elicit and intensify embodiment perception during MVF (Li et al., 2024). However, this protocol was impractical in primary care hospitals and home settings due to equipment limitations.

Rubber hand illusion (RHI) and action observation (AO) are commonly used paradigms in the field, which are both driven by visual inputs and influence the cognitive process via multisensory integration (Castro and Schenke, 2024; Horvath et al., 2020). RHI is a standard model for studying body ownership (one of the components of embodiment) (Costantini and Haggard, 2007), and person in RHI trails would easily report a rubber hand is part of his/her body while seeing synchronous brushing of the rubber hand and their own hidden hand (Botvinick and Cohen, 1998). This perceptual illusion is generated by integrating the visual, tactile, and proprioceptive inputs, which has been reported to be associated with the activation of motor cortex, ventral PMC, PPC, and insula (Botvinick and Cohen, 1998; Golaszewski et al., 2021). Llorens et al. (2017) employed the RHI paradigm to demonstrate enhanced body ownership and action in stroke patients, potentially from visual dominance over proprioception and supplementary motor area in promoting body schema plasticity. Valadez-Roque et al. (2024) combined the RHI with TMS-EEG demonstrated that the perception of bodily ownership can be achieved by remodeling the alpha band functional connectivity in the primary somatosensory cortex (Pisoni et al., 2025). MVF and RHI contain same sensory inputs and activate overlapping brain areas, implying shared neural networks of these two interventions. Regarding to AO, it plays a critical role in motor relearning after stroke, which involved the use of videos or other means to allow persons observe and imitate specific motor tasks. Harmsen et al. (2015) conducted a randomized controlled trial specifically examining an AO programmed based on mirror therapy, demonstrating that mirror therapy-based AO protocol promotes motor learning following stroke. Some researchers proposed that AO acts as a visual stimulus, which helps individuals generate a sense of kinesthesia by visual signals (Sim et al., 2015). This cognitive process is associated with the activation of MNS, similar with MVF (Savaedi et al., 2025). Moreover, some studies reported the effect of AO on embodiment alterations (Fabbri-Destro and Rizzolatti, 2008; Molenberghs et al., 2012; Campbell et al., 2021; Holper et al., 2010; Ang et al., 2023).

As mentioned above, these two approaches, RHI and AO, are efficient paradigms to investigate embodiment perception. Although, RHI primarily engages the sensorimotor network and AO predominantly targets MNS, they share similarities in cognitive processes grounded in multisensory integration and activate overlapping neural networks with MVF (Sakamoto and Ifuku, 2021; Hardwick et al., 2018). Thus, we aimed to investigate the behavioral effects of RHI/AO priming on subsequent embodiment perception during MVF. In this study, RHI and AO were employed as pre-activation paradigms to investigate effects of neural priming, and subsequent MVF trainings were conducted, including simple motor tasks (SMT) and object-based tasks (OBT). Behavioral evaluation and queries were employed to compare the priming effect. Additionally, machine learning (random forest, RF) was used to assess relative variable importance as key factors which can influence the experience of embodiment. Our hypothesis was that priming with RHI and AO paradigms could improve embodiment perception during subsequent MVF. Furthermore, we hypothesized that motor tasks with objects could promote the embodiment occurrences during MVF. This proof-of-concept study would explore behavioral outcomes that could guide approaches to promote embodiment perception during MVF paradigms, which could form the basis for larger, more rigorous clinical studies.

## 2 Method

### 2.1 Study design and participants

**Figure 1:**
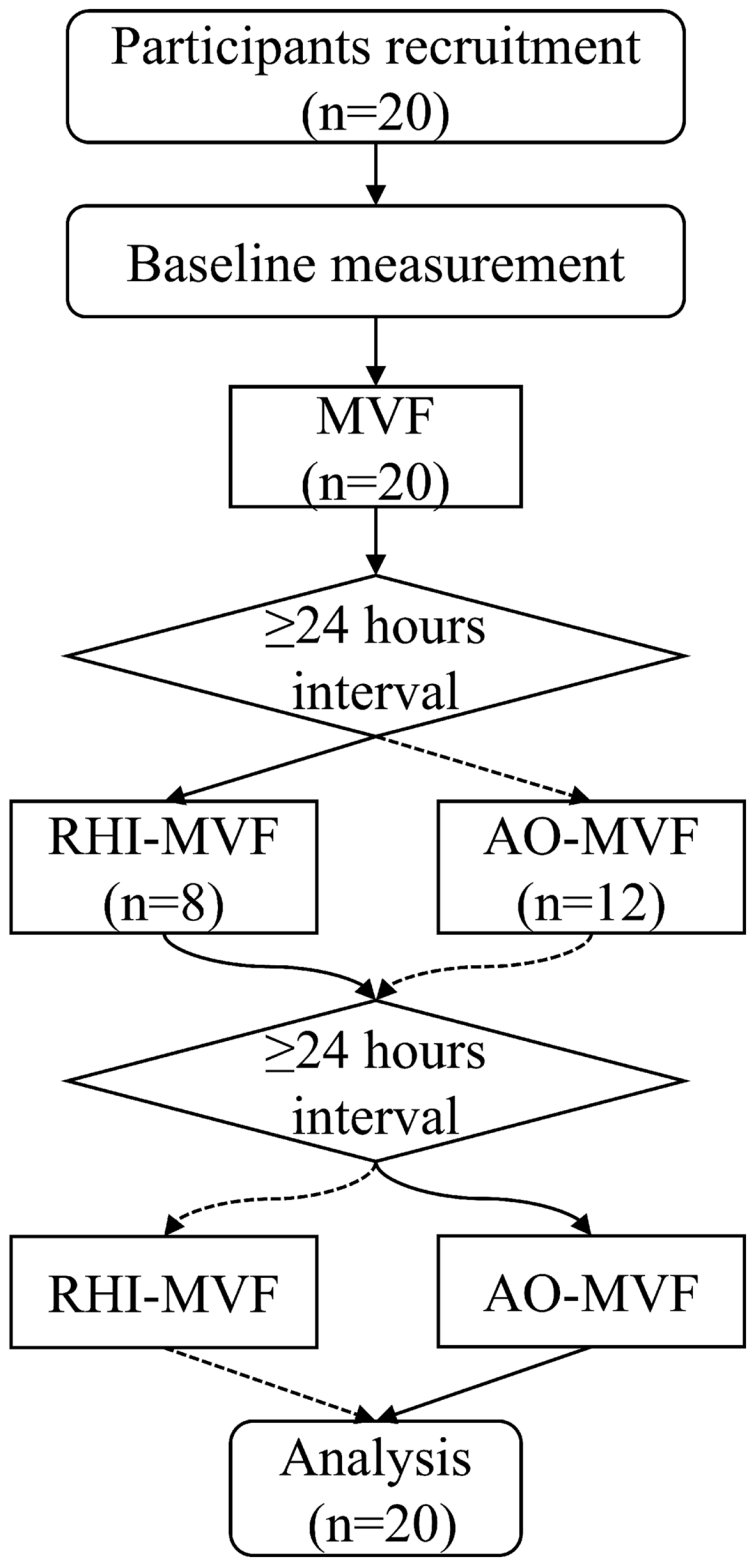
Flow diagram of the randomized, observational crossover study for mirror visual feedback involved network priming on embodiment perception in healthy subjects.

This was an intergroup randomized, observational crossover study, which aimed to compare the effects of different priming strategies using AO or RHI on embodiment perception due to subsequent MVF, as shown in Figure 1. A total of 20 participants were recruited to account for potential dropouts. From May 19th to May 30th, 2025, 20 healthy participants (age: 22.50 ± 3.80 years; 13 females, 7 males; BMI: 22.51 ± 7.38 kg/m^2^; all right-handed) were recruited.

The healthy participants who met the following criteria were enrolled: (1) aged between 20 and 35 years; (2) right-handed (assessed via the Edinburgh Handedness Inventory, with the score over 40); (3) none of the participants had participated in any MVF studies or experiments in advance; (4) normal vision or corrected to normal vision. Who met any of the following conditions were excluded: (1) received non-invasive brain stimulation in the past 3 months, such as rTMS or transcranial direct current stimulation (tDCS); (2) have physical or mental problems which affected the attending of the experiment; (3) use of sedative-hypnotics within the past 3 months. All the participants were informed of the objectives and procedures of this study and signed informed consents approved Tongji University Review Boards (tjdxsr2024098) prior to enrollment.

### 2.2 Experimental paradigm

**Figure 2:**
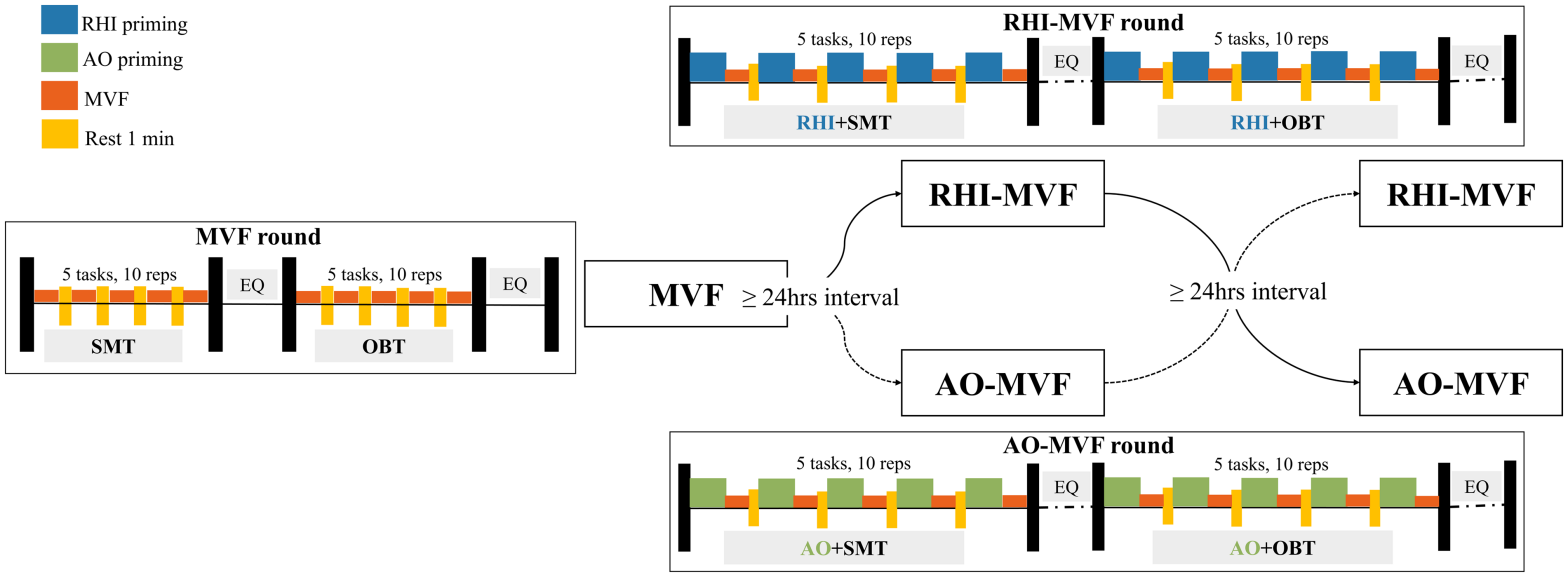
Demonstration of the experimental protocol.

This experiment contained three rounds: MVF, RHI-MVF, and AO-MVF like Figure 2. At first, all the participants completed the round of MVF, and after 24 h, they received the round of RHI-MVF or AO-MVF at a random order with an interval of 24 h. During pre-activation paradigm, the intermittent between RHI/AO and MVF was required within 15 s. Each round comprised two sessions, including session of SMT and session of OBT. In addition, each session contained 5 tasks, which was repeated 10 times at a frequency of 2 s per time. The degree of embodiment perception was assessed after each task and the embodiment questionnaire (EQ) was administered after each session.

#### 2.2.1 Round of MVF

**Figure 3:**
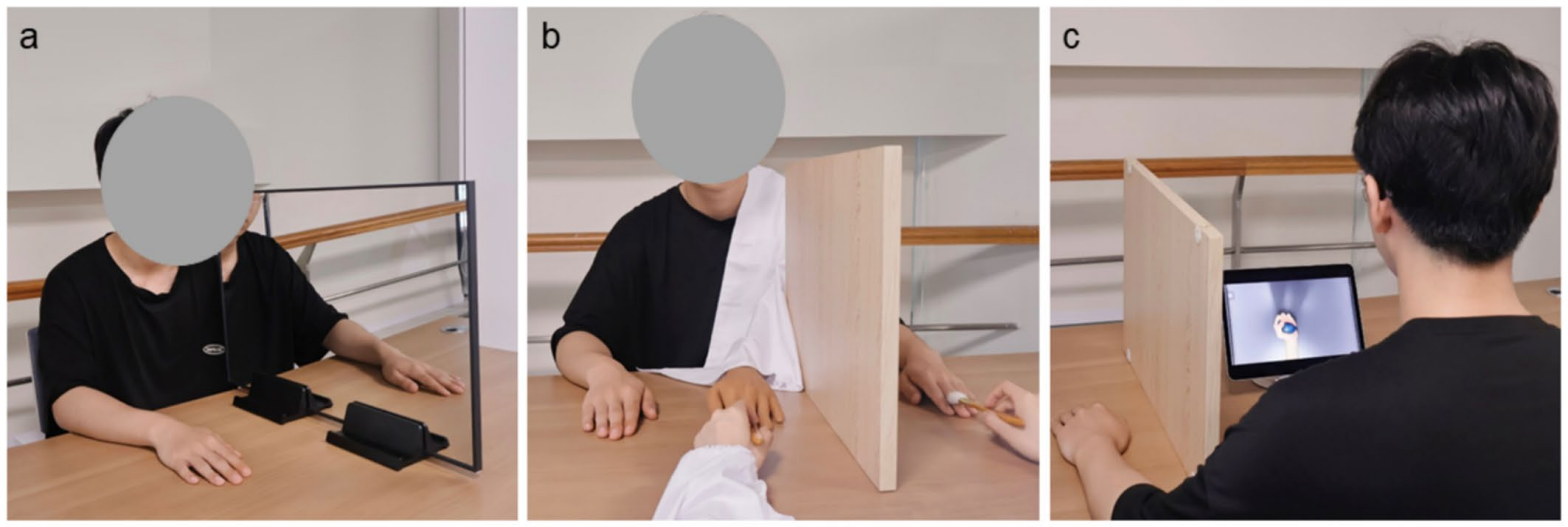
Diagrammatic drawing of MVF **(a)**, RHI priming **(b)**, and AO priming **(c)**.

In the round of MVF, a plain mirror (40 cm × 60 cm) was positioned on a table in the sagittal plane of participants to provide MVF. Participants sat on a height-adjustable chair and put their upper limbs on the table, where the mirror was placed in the middle of both limbs. In this study, the participant’s right limb was placed on the reflective side of the mirror, while left limb was placed on the back of the mirror and remained stationary during tasks like Figure 3a and Supplementary Video.

During this round, participants performed the tasks of SMT and OBT under pure MVF, and they were instructed to convince themselves that their left hands were moving and to promptly report the researcher when they had a sense of embodiment.

#### 2.2.2 Round of RHI-MVF

In the RHI-MVF round, participants received the procedure of RHI as a pre-activation paradigm and then conducted tasks of SMT and OBT under pure MVF, of which the operations were the same as the round of MVF. In the RHI pre-activation phase, a board was used to block the vision of left upper limb and a rubber hand (26 cm × 6 cm) was placed in the right side of the board, where a white cloth was used to cover the gap between the rubber hand and the participant’s trunk like Figure 3b and Supplementary Video. During RHI, participants received synchronous brushing of the rubber hand and their left hand, which lasted for 1 min before each task of SMT and OBT under MVF in two sessions.

#### 2.2.3 Round of AO-MVF

In the AO-MVF round, pre-recorded videos of left-hand performing tasks of SMT and OBT were employed to provide the AO pre-activation via a 12-inch tablet, like Figure 3c and Supplementary Video. Similar with the procedure of RHI pre-activation paradigm, participants were required to watch the videos of tasks of SMT and OBT for 1 min before conducting responding tasks under MVF.

#### 2.2.4 Session of SMT

Five tasks were contained in the SMT session, which specifically focused on basic physiological movements involving arm, wrist, hand, and thumb. The tasks of SMT included fist clenching (SMT-1), thumb-to-index finger opposition (SMT-2), lateral pinch with the thumb (SMT-3), dorsal wrist extension (SMT-4), and forearm rotation (SMT-5) (see Supplementary Video).

#### 2.2.5 Session of OBT

Five tasks were included in the session of OBT, which integrated basic motor trainings with therapeutic assistive devices, corresponding to SMT and emphasizing task-oriented movements with tactile inputs. The OBT session incorporated five tasks: squeezing a 5-pound grip ball (7 cm) (OBT-1), grasping a wooden cube (3 cm × 3 cm × 3 cm) (OBT-2), picking up a paper clip (2.8 cm) (OBT-3), gripping a wooden stick (10 cm × 1.8 cm) (OBT-4), and flipping a card (5.7 cm × 8.7 cm) (OBT-5) (see Supplementary Video).

### 2.3 Outcome measure

#### 2.3.1 Latency time and number of embodiment occurrences

Latency time (LT) was defined as the time taken from the beginning of each trial of a motor task to the time point when the sense of embodiment appeared. According to our previous studies, the upper limit of LT in this present study was set to 20 s, which would contribute to investigate the priming effects on embodiment alterations (Li et al., 2024; Ding et al., 2020a). If participants perceived embodiment in less than 20 s, this trial was marked as a successful trial and the actual time taken was recorded as LT. However, the LT was recorded as 20 s for unsuccessful trials. Number of embodiment occurrences was defined as the trials with successful embodiment perception out of 10 times in each task.

#### 2.3.2 Degree of embodiment perception

To generally evaluate the embodiment perception of each task, participants were required to rate how strong the embodiment perception after one task via an 11-item Likert scale, where “−5” represented “strongly disagree” with the statement, and “+5” represented “strongly agree.” The survey was conducted for each task, totally 10 times (5 tasks/session *2 sessions).

#### 2.3.3 Embodiment questionnaire

To assess the intensity of embodiment and to compare the effects of three rounds and two sessions on the perception of embodiment, EQ was employed after each session based on previous studies (Botvinick and Cohen, 1998; Ding et al., 2020a). Our previous research has successfully applied this questionnaire in both healthy and patient populations, validating its effectiveness in detecting the enhancement of embodiment perception, the promotion of facial embodiment, and the regulation via rTMS (Li et al., 2024; Ding et al., 2020a, 2020b). These results supported the reliability and sensitivity of the EQ in assessing embodiment experiences. EQ contained symmetry of the left hand reflection (S-1 “the left hand seemed natural,” S-2 “the left hand seen in the mirror was superimposed with the limb behind the mirror”), ownership of the hand (O-1 “it felt like the left hand I saw in the mirror was my real left limb,” O-2 “it seemed the left hand in the mirror was part of my body”), agency of reflection (A-1 “it felt like my left hand was moving with the image in the mirror,” A-2 “it felt as if I could control the movement of the left hand in the mirror”), and deafference (D-1 “it seemed hard for me to discern the location of my left hand,” D-2 “the left hand in the mirror seemed unusual”). Participants were required to rate each statement after each session of different rounds via an 11-item Likert scale, where “-5” represented “strongly disagree” and “+5” indicated “strongly agree” with the statement.

### 2.4 Data analyses

#### 2.4.1 Medical statistical analysis

Statistical analyses were performed using SPSS 26.00 software. The Shapiro–Wilk test was used to assess normality, and the Levene’s test was used to assess the variances. The sequence effect was examined with an Independent Samples *t*-test or Mann–Whitney *U* test immediately after the third round of intervention, expressed in *p*-values (Lin et al., 2025). The carryover effect was examined with the independent samples *t*-test or Mann–Whitney *U* test to compare for the differences between the two pre-activation paradigms, including RHI-MVF and AO-MVF, expressed in *p*-values (Wellek and Blettner, 2012).

The average scores of each EQ statement and number of embodiment occurrences for two sessions were compared using the Friedman test within three rounds. *Post-hoc* analyses were made using the Wilcoxon signed-rank test, with Bonferroni corrections. The Wilcoxon signed-rank test was applied to compare the average EQ and number of embodiment occurrences within SMT and OBT of three rounds. The average of LTs for two sessions were compared within three rounds (MVF vs. RHI-MVF vs. AO-MVF), using one-way analysis of variance (ANOVA). Moreover, paired sample *t*-test was used to compare the average LTs within SMT and OBT of three rounds. Data with normal distribution were expressed as mean ± standard deviation and others were expressed as quartiles. Two-tailed tests were conducted with a significance level set at 0.05.

#### 2.4.2 Variable importance for RF

The RF algorithm was employed to assess the impact of various features on embodiment perception by the PyCharm Community Edition 2024.3.1 software, via the variable importance measures (VIM) to evaluate contribution of interventions to the model’s performance. The dataset was divided into a train set (80%) and a test set (20%). The evaluated features included four interventions such as RHI priming, AO priming, SMT session and OBT session, with the degree of embodiment perception of each task and EQ scores of each session serving as the target variables. The evaluated variables were processed by one-hot encoding, where a value of 1 was assigned if the pattern existed within a given trial or session, and 0 otherwise. For the target variables, the original values within the range of [−5, 5] were discretized into three categories: values greater than 0 were assigned a value of 1, values less than 0 were assigned a value of −1, and values equal to 0 were assigned a value of 0. This discretization simplifies the target space while retaining key directional information, aiding the model learning (Cascella et al., 2024).

VIM was evaluated based on mean square error (MSE) variations reflecting each feature’s contribution to the model’s performance. MSE measures a model’s predictive performance by calculating the mean of the squared differences between predicted and actual values (Breiman, 2001). The MSE is computed as:


$$\mathrm{MSE}=\frac{1}{n}\sum_{i=1}^{n}{\left(y_{i}-{\hat{y}}_{i}\right)}^{2}$$

where *n* is the sample size, $y_{i}$ is the true value of the i-th sample, ${\hat{y}}_{i}$ is the predicted value for the *i*-th sample. A higher VIM score indicates a greater contribution of the feature to the classification model. Model performance in test set was evaluated using accuracy, precision, recall and *F*_1_-score, with values closer to 1 indicating better predictive performance.

## 3 Results

Twenty participants were recruited in the study. All the participants successfully completed three rounds and questionnaires without any adverse events. The analysis showed no significant sequence effects (*p*-values = 0.151–1.00). The carryover effect was determined to be non-significant (*p*-values = 0.132–0.938).

### 3.1 Efficacy of EQ

#### 3.1.1 Results of the EQ for three rounds

**Figure 4:**
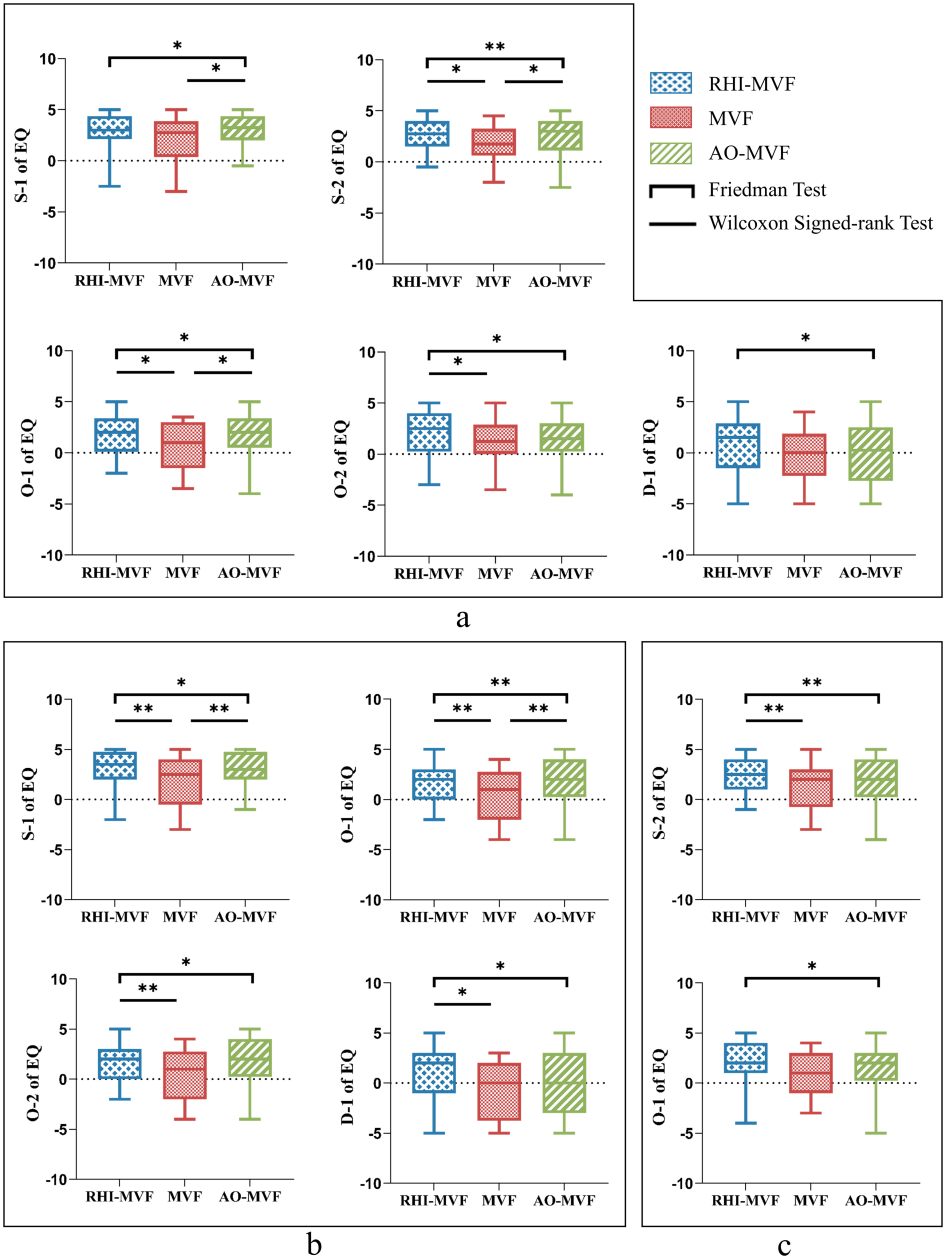
Results of the EQ. **(a)** The results of the Friedman test and further Wilcoxon signed-rank test on the EQ within three rounds. **(b)** The results of the Friedman test and further Wilcoxon signed-rank test on the EQ for three rounds in SMT session. **(c)** The results of the Friedman test and further Wilcoxon signed-rank test on the EQ for three rounds in OBT session; ^*^p < 0.05 and ^**^p < 0.01.

The results of the Friedman test on EQ of three rounds were presented in Table 1. Further Wilcoxon signed-rank test after Bonferroni corrections demonstrated that EQ was significantly greater in the RHI-MVF and the AO-MVF than in the MVF round for five statements (S-1: AO-MVF vs. MVF, *Z* = 2.818, *r* = 0.630, *p* = 0.015; S-2: RHI-MVF vs. MVF, *Z* = 2.539, *r* = 0.568, *p* = 0.033; O-1: RHI-MVF vs. MVF, *Z* = 2.513, *r* = 0.562, *p* = 0.036, AO-MVF vs. MVF, *Z* = 2.489, *r* = 0.556, *p* = 0.039; O-2: RHI-MVF vs. MVF, *Z* = 2.598, *r* = 0.581, *p* = 0.027; D-1: no significant differences between the pairwise comparisons), which indicated that RHI-MVF or AO-MVF paradigms had the capability in inducing embodiment perception, as shown in Figure 4a. In addition, the Mann–Whitney *U* test showed that RHI-MVF paradigm performed better on the ownership subscale in the EQ than AO-MVF paradigm (O-1: *Z* = 2.020, *r* = 0.319, *p*-values = 0.043; O-2: *Z* = 2.073, *r* = 0.328, *p*-values = 0.038).

**Table 1:** Results of the Friedman test on EQ for MVF, RHI-MVF and AO-MVF within three rounds.

EQ	MVF	RHI-MVF	AO-MVF	$\chi^{2}$	Kendall’s *W*	*p*
S-1	2.75 [0.38, 3.88]	3.00 [2.13, 4.38]	3.25 [2.00, 4.38]	7.304	0.183	0.026^*^
S-2	1.75 [0.63, 3.25]	2.75 [1.50, 4.00]	3.00 [1.13, 4.00]	9.973	0.249	0.007^**^
O-1	1.00 [−1.50, 3.00]	2.00 [0.13, 3.38]	2.00 [0.50, 3.38]	8.351	0.209	0.015^*^
O-2	1.25 [0.00, 2.88]	2.50 [0.25, 4.00]	1.50 [0.25, 3.00]	8.899	0.222	0.012^*^
A-1	1.75 [0.63, 3.50]	2.50 [1.13, 3.38]	2.50 [1.00, 3.88]	0.812	0.020	0.666
A-2	2.75 [0.00, 4.00]	2.50 [1.13, 4.00]	2.50 [0.00, 3.50]	4.899	0.122	0.086
D-1	0.00 [−2.25, 1.88]	1.50 [−1.50, 2.88]	0.25 [−2.75, 2.50]	7.373	0.184	0.025^*^
D-2	0.50 [−3.63, 2.38]	2.00 [−2.25, 2.88]	1.50 [−0.75, 2.50]	0.724	0.018	0.696

^*^p < 0.05 and ^**^p < 0.01.

#### 3.1.2 Results of EQ for three rounds in two sessions

The results of the Friedman test on EQ for three rounds in SMT were presented in Figure 4b and Supplementary Data 1. Further Wilcoxon signed-rank test after Bonferroni corrections demonstrated that EQ was significantly greater in the RHI-MVF and the AO-MVF than MVF round in the session of SMT for four question (S-1: RHI-MVF vs. MVF, *Z* = 2.674, *r* = 0.598, *p* = 0.024, AO-MVF vs. MVF, *Z* = 2.970, *r* = 0.664, *p* = 0.009; O-1: RHI-MVF vs. MVF, *Z* = 2.766, *r* = 0.618, *p* = 0.018, AO-MVF vs. MVF, *Z* = 3.143, *r* = 0.703, *p* = 0.006; O-2: RHI-MVF vs. MVF, *Z* = 2.754, *r* = 0.616, *p* = 0.018; D1: RHI-MVF vs. MVF, *Z* = 2.473, *r* = 0.553, *p* = 0.039), which indicated that RHI-MVF or AO-MVF paradigms had the capability in inducing embodiment perception. However, the independent samples *t*-test and Mann–Whitney *U* test showed that no significant results were obtained between RHI-MVF and AO-MVF in the SMT session (*p*-values > 0.05).

The results of the Friedman test on EQ for three rounds in OBT are presented in Figure 4c and Supplementary Data 1. Further Wilcoxon signed-rank test after Bonferroni corrections demonstrated that EQ was significantly greater in the RHI-MVF and the AO-MVF than MVF in the session of OBT for four question (S-2: RHI-MVF vs. MVF, *Z* = 3.051, *r* = 0.682, *p* = 0.006; O-1: no significant differences between the pairwise comparisons), which indicated that RHI-MVF or AO-MVF paradigms had the capability in inducing embodiment perception. Notably, in the OBT session, the Mann–Whitney *U* test showed that RHI-MVF paradigm outperformed the AO-MVF paradigm on EQ items O-1, O-2, and A-1 (O-1: *Z* = 2.282, *r* = 0.361, *p*-values = 0.025; O-2: *Z* = 2.084, *r* = 0.330, *p*-values = 0.039; A-1: *Z* = 2.299, *r* = 0.363, *p*-values = 0.025).

#### 3.1.3 Results of EQ for two sessions

The Wilcoxon signed-rank test revealed that there was no statistically significant difference between SMT and OBT on the EQ (see Supplementary Data 2).

### 3.2 Efficacy of the LT and number of embodiment occurrences

#### 3.2.1 Results of LT, number of embodiment occurrences for three rounds and in two sessions

There were no significant differences observed in comparisons for three rounds and in two sessions (see Supplementary Data 3).

#### 3.2.2 Results of LT, number of embodiment occurrences for two sessions (SMT vs. OBT)

The average LT, number of embodiment occurrences for three rounds were calculated within two sessions. Paired sample *t*-tests demonstrated that the LT and number of embodiment occurrences of OBT was significantly than SMT as shown in Table 2. Critically, the average LT in OBT was significantly longer than in SMT (*t* = −2.788, Cohen’s *d* = −0.623, *p* = 0.012). Additionally, there were no significant differences observed in other comparisons for two sessions.

**Table 2:** Results of the paired sample *t*-tests on LT, number of embodiment occurrences for SMT and OBT sessions.

Outcomes	SMT	OBT	*t*	Cohen’s *d*	*p*
LT					
Average	7.43 ± 2.47	8.49 ± 2.89	−2.788	−0.623	0.012^*^
Task-1	7.56 ± 2.40	7.43 ± 2.47	0.405	0.091	0.690
Task-2	8.08 ± 2.58	9.75 ± 0.43	−2.686	−0.601	0.015^*^
Task-3	8.71 ± 1.76	7.34 ± 3.02	2.422	0.542	0.026^*^
Task-4	7.61 ± 2.70	8.66 ± 3.12	−1.756	−0.393	0.095
Task-5	8.89 ± 1.12	8.23 ± 1.51	2.074	0.464	0.052
Number of embodiment occurrences					
Average	9.66 ± 0.43	9.59 ± 0.55	0.555	0.124	0.585
Task-1	9.70 ± 0.43	9.66 ± 0.43	0.496	0.111	0.625
Task-2	9.45 ± 0.85	7.06 ± 3.03	3.091	0.691	0.006^**^
Task-3	8.08 ± 2.58	9.75 ± 0.43	−2.686	−0.601	0.015^*^
Task-4	7.35 ± 2.55	7.61 ± 2.70	−2.149	−0.480	0.045^*^
Task-5	7.61 ± 2.70	7.43 ± 2.47	0.872	0.195	0.394

^*^p < 0.05 and ^**^p < 0.01.

### 3.3 The VIM of interventions influencing the degree of embodiment perception and EQ

**Figure 5:**
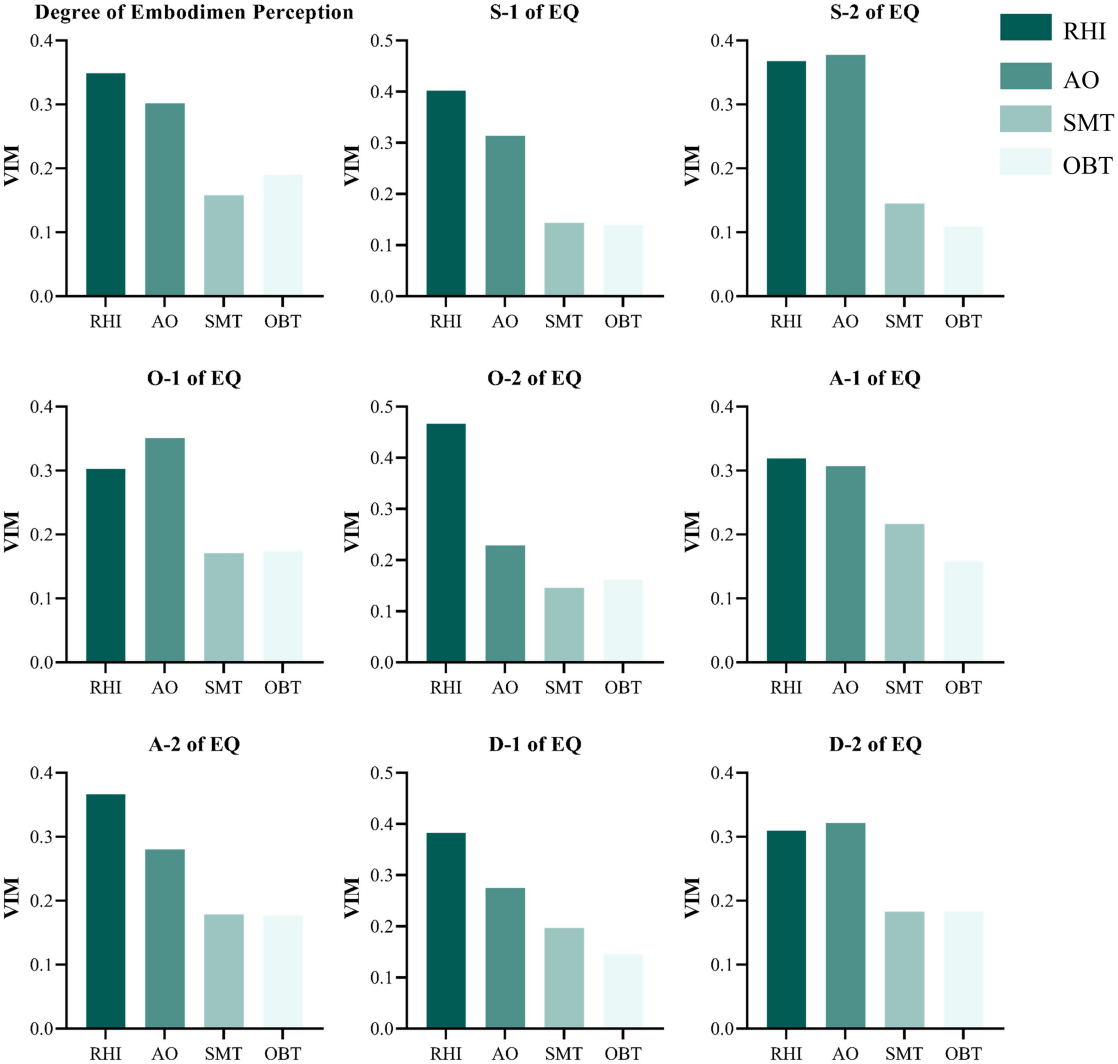
The VIM of interventions influencing the degree of embodiment perception and EQ.

Nine models were trained to assess the VIM of features influencing degree of embodiment perception and EQ scores by RF algorithm (see Figure 5).

A total of 480 the degree of embodiment perception data points was allocated to the training set, and 120 data points were allocated to the testing set. The VIM for degree of embodiment perception was as follows: RHI = 0.349, AO = 0.302, OBT = 0.191 and SMT = 0.158. The accuracy of model test set achieved was 92.50%. For the EQ, 96 questionnaires were used for training, and 24 questionnaires were used for testing. In S-1, O-2, A-1, A-2, and D-1, the RHI priming showed the highest contributions (VIM: 0.319–0.467). In S-2, O-1 and D-2, the highest contribution was AO priming (VIM: 0.322–0.378). Additionally, the model’s test set accuracy, precision, recall and *F*_1_-score exhibits polarization, with S-1, S-2, O-1, O-2, A-1, A-2 (accuracy: 79.17%–87.50%, precision: 62.67%–85.56, recall: 75.00%–92.50%, *F*_1_-score: 69.96%–88.90%) performing well, while the D-1 (accuracy = 41.67%, precision = 34.90%, recall = 41.67%, *F*_1_-score = 37.78%) and D-2 (accuracy = 50.00%, precision = 41.67%, recall = 50.00%, *F*_1_-score = 45.30%) demonstrated lower performance (see Supplementary Data 4).

## 4 Discussion

As a proof-of-concept study, our results presented behavioral evidence that the MVF involved networks priming via RHI/AO could strengthen the intensity of embodiment perception during subsequent MVF, which might enhance therapeutic effect via reducing the variation of embodiment perceiving among individuals. Moreover, machine learning analysis further indicated the relative importance of pre-activation paradigms (RHI >AO) in intensifying embodiment perception, rather than types of motor tasks during MVF. On the other hand, our results showed that motor tasks with objects could promote the elicitation of embodiment due to MVF. This study suggested potential strategies to upregulate the receptiveness to MVF in neuro-rehabilitation.

In this study, we found that the intensity of embodiment perception during MVF was facilitated with either RHI or AO priming. As a classic paradigm, RHI generated a perceptual illusion, which combined multimodal sensory information to adjust body perception (Sonobe et al., 2023). Our study employed the perceptual illusion to rehearse the alteration of bodily consciousness of left limb. This might be one possible interpretation for the strengthened embodiment perception during MVF after RHI priming. Moreover, studies reported the activation of sensorimotor network during RHI, including motor cortex, ventral PMC, PPC, and insula, which was overlapped with MVF (Li et al., 2024; Seiyama et al., 2024; Lira et al., 2018). Thus, our study used RHI to pre-activate the shared sensorimotor network, which upregulated the response of the participants’ brain and resulted in strengthened embodiment perception during subsequent MVF. As for the AO pre-activation paradigm, the visual observation was used to prime mirror neurons in this study, such as PMC and STG, of which the involvement was also obtained during MVF in previous studies (Buccino et al., 2006; Zhang et al., 2018; Pomeroy et al., 2005; Grezes and Decety, 2001). Thus, AO pre-activated MNS for subsequent MVF and contributed to facilitate embodiment perception of MVF, as suggested by our results. Moreover, studies have reported the ability of AO in improving motor learning, specifically the first-person perspective AO (Yu and Park, 2022). In this study, the strengthening effect of AO on embodiment perception during MVF might be attributed to the visual inputs of left-hand action, which potentially enhanced the bodily awareness and attentional source to left limb (Dufour and Touzalin, 2008; Bridgeman and Tseng, 2011; Ding et al., 2024). These findings supported our hypothesis that priming of MVF involved networks could facilitate subsequent embodiment perception.

In addition, this present study firstly applied machine learning analysis to quantitatively investigate interventional factors influencing the embodiment perception of MVF. According to the results of VIM using RF algorithm, pre-activation paradigms are important factors relevant to intensifying embodiment perception during MVF, where the paradigm of RHI priming was pronounced to enhance the degree of embodiment. One possible explanation might be that while having perceptual illusion generated by RHI, participants would embody the rubber hand as their own hand, which might benefit for perceiving embodiment of MVF (Isayama et al., 2019; Ehrsson et al., 2008). On the other hand, the broader pre-activation of brain regions after RHI might be another reason for distinct behavior performance of MVF (Huang et al., 2024).

However, no effect of RHI/AO priming on the elicitation of embodiment perception due to MVF was observed in our study, as suggested by the unsignificant changes of LT and number of embodiment occurrences among three rounds. Studies have reported that embodiment perception of MVF was generated via sensory integration, where multisensory inputs and long-term stimulation were important for the elicitation (Ding et al., 2020a; Ding et al., 2023). However, the priming paradigms emphasized the pre-activation of MVF involved networks, rather than the process of sensory integration, which might interpret our results. This was also supported by the comparison between two types of motor tasks, where longer average LT and the increased number of embodiment occurrence in part of motor tasks were obtained in the OBT session, comparing to the session of SMT.

The present study had several limitations. First, the average age of the participants was relatively young. Future research should aim to recruit elderly individuals or patients suffering from phantom limb pain or hemiparesis. Second, although this is a proof-of-concept study, the relatively small number of participants still limited the analysis power of the study. In the future, we plan to expand the dataset and explore alternative algorithms to improve the adaptability to small sample size. Moreover, the EQ has been used in our previous studies, but future research should incorporate electrophysiological indicators or neuroimaging techniques to assess the effect of MVF involved network priming on the changes of cortical excitabilities related to embodiment perception.

## 5 Conclusion

The present study firstly showed that both RHI and AO based pre-activations of MVF involved networks could strengthen the intensity of embodiment perception during MVF in healthy participants. Further analysis using machine learning supported the importance of RHI/AO priming in intensifying embodiment, and suggested that RHI priming counted for more. In addition, our results proposed that OBT contributed to elicit embodiment due to MVF. This study provided alternative strategies to upregulate embodiment perception of MVF including elicitation and intensification, and suggested to combine RHI/AO priming with object-based motor tasks as a superior protocol of MVF in future clinical design.

## Data Availability

The raw data supporting the conclusions of this article will be made available by the authors, without undue reservation.
