# Supplementary material for: The effects of mirror visual feedback involved network priming on embodiment perception in healthy subjects: a proof-of-concept study
Source: Front Neurosci. 2026 Mar 12;20:1781002. doi: 10.3389/fnins.2026.1781002 (PMC13018146; doi:10.3389/fnins.2026.1781002)
Supplement: Supplementary file 1 [file Table_1.DOCX]

**Supplementary Data**

**Data 1** Results of the Friedman test on EQ for three rounds in two sessions.

| **EQ** | **MVF** | **RHI-MVF** | **AO-MVF** | $\mathbf{x}^{\mathbf{2}}$ | ***Kendall's W*** | ***p*** |
| --- | --- | --- | --- | --- | --- | --- |
| **SMT session** |  |  |  |  |  |  |
| S-1  S-2  O-1  O-2  A-1  A-2  D-1  D-2 | 2.50[-0.50, 4.00] | 3.50[2.00, 4.75] | 3.00[2.00, 4.75] | 8.667 | 0.217 | 0.013* |
|  | 2.00[1.00, 3.75] | 3.00[1.25, 4.00] | 3.50[1.00, 4.00] | 2.030 | 0.051 | 0.362 |
|  | 1.00[-2.00, 2.75] | 2.00[0.00, 3.00] | 2.00[0.25, 4.00] | 13.303 | 0.333 | 0.001** |
|  | 1.50[-1.00, 2.75] | 3.00[1.00, 4.00] | 2.00[0.25, 4.00] | 4.804 | 0.120 | 0.015* |
|  | 2.00[1.00, 3.00] | 2.00[1.00, 3.00] | 3.00[1.00, 4.00] | 0.034 | 0.001 | 0.983 |
|  | 3.00[-0.50, 4.00] | 3.00[1.25, 4.00] | 2.50[-1.25, 4.00] | 2.030 | 0.051 | 0.362 |
|  | 0.00[-3.75, 2.00] | 0.00[-1.00, 3.00] | 0.00[-3.00, 3.00] | 6.536 | 0.163 | 0.038* |
|  | 0.00[-5.00, 2.75] | 2.00[-2.50, 3.00] | 1.00[-1.50, 3.00] | 4.456 | 0.111 | 0.108 |
| **OBT session** |  |  |  |  |  |  |
| S-1  S-2  O-1  O-2  A-1  A-2  D-1  D-2 | 2.00[1.25, 4.00] | 3.00[2.00, 4.00] | 3.00[2.00, 4.00] | 5.887 | 0.147 | 0.053 |
|  | 2.00[-0.75, 3.00] | 2.50[1.00, 4.00] | 3.00[2.00, 4.00] | 11.893 | 0.297 | 0.003** |
|  | 1.00[-1.00, 3.00] | 2.00[2.00, 4.00] | 2.00[0.25, 3.00] | 11.108 | 0.278 | 0.004** |
|  | 1.00[0.25, 2.75] | 2.00[1.00, 4.00] | 1.50[0.25, 2.75] | 3.500 | 0.088 | 0.174 |
|  | 2.50[0.00, 4.00] | 2.50[1.25, 4.00] | 2.00[1.25, 3.75] | 1.138 | 0.028 | 0.566 |
|  | 2.00[1.00, 4.00] | 2.00[1.00, 4.00] | 2.50[1.25, 3.75] | 2.355 | 0.059 | 0.308 |
|  | 0.00[-2.00, 3.00] | 1.00[-2.00, 3.00] | 0.50[-2.75, 2.00] | 5.727 | 0.143 | 0.057 |
|  | 1.50[-3.75, 2.00] | 2.00[-2.50, 3.00] | 2.00[-1.50, 3.00] | 1.412 | 0.035 | 0.494 |

*：*p*＜0.05; **：*p*＜0.01.

**Data 2** **Results of the Wilcoxon signed-rank test on EQ for SMT and OBT Sessions.**

| **Outcomes** | **SMT** | **OBT** | **Z** | ***p*** |
| --- | --- | --- | --- | --- |
| S-1 | 3.00[1.333, 4.59] | 2.67[2.00, 3.92] | 0.340 | 0.734 |
| S-2 | 2.67[1.42, 3.59] | 2.67[0.50, 3.33] | 1.090 | 0.187 |
| O-1 | 1.16[-0.51, 3.25] | 1.67[0.08, 3.00] | 1.319 | 0.187 |
| O-2 | 1.33[0.33, 3.59] | 1.50[0.50, 2.92] | 0.597 | 0.551 |
| A-1 | 2.33[0.75, 3.59] | 2.84[1.08, 3.33] | 0.214 | 0.831 |
| A-2 | 3.00[-0.08, 4.00] | 3.00[0.42, 3.59] | 0.356 | 0.722 |
| D-1 | 0.33[-1.42, 2.25] | 0.17[-1.59, 1.92] | 0.363 | 0.717 |
| D-2 | 0.67[-2.17, 2.59] | 1.34[-1.25, 2.59] | 0.598- | 0.550 |

**Data 3** Results of LT，Number of Embodiment Occurrences for Three Rounds and in Two Sessions.

| **Outcomes** | **MVF** | **RHI-MVF** | **AO-MVF** | **F/**$\mathbf{x}^{\mathbf{2}}$ | ***p valve*** |
| --- | --- | --- | --- | --- | --- |
| **LT** |  |  |  |  |  |
| three rounds | 7.69±2.31 | 8.10±3.18 | 8.10±3.22 | 0.133 | 0.875 |
| SMT session | 7.25±2.05 | 7.50±3.34 | 7.55±3.35 | 0.058 | 0.944 |
| OBT session | 8.12±2.96 | 8.70±3.31 | 8.65±3.48 | 0.196 | 0.822 |
| **Number of Embodiment Occurrences** |  |  |  |  |  |
| three rounds | 9.75[9.60, 9.98] | 9.90[9.45, 10.00] | 9.85[9.45, 10.00] | 0.259 | 0.878 |
| SMT session | 9.80[9.60, 10.00] | 10.00[9.80, 10.00] | 10.00[9.65, 10.00] | 1.244 | 0.537 |
| OBT session | 9.80[9.60, 10.00] | 10.00[9.40, 10.00] | 10.00[9.40, 10.00] | 0.298 | 0.862 |

**Data 4** The evaluation performance of nine models in test set, %.

| **Outcomes** | **Accuracy** | **Precision** | **Recall** | **F1 Score** |
| --- | --- | --- | --- | --- |
| Degree of embodiment perception | 92.50 | 85.56 | 92.50 | 88.90 |
| **EQ** |  |  |  |  |
| S-1 | 87.50 | 76.56 | 87.50 | 81.67 |
| S-2 | 87.50 | 76.56 | 87.50 | 81.67 |
| O-1 | 75.00 | 75.39 | 75.00 | 74.04 |
| O-2 | 83.33 | 69.44 | 83.33 | 75.76 |
| A-1 | 87.50 | 76.56 | 87.50 | 81.67 |
| A-2 | 79.17 | 62.67 | 79.17 | 69.96 |
| D-1 | 41.67 | 34.90 | 41.67 | 37.78 |
| D-2 | 50.00 | 41.67 | 50.00 | 45.30 |
